# Assessing the impact of the COVID-19 pandemic on routine childhood vaccination uptake, the Netherlands, 2016 to 2025

**DOI:** 10.2807/1560-7917.ES.2026.31.40.2600159

**Published:** 2026-10-08

**Authors:** Joyce Pijpers, Manon Haverkate, Ruben van Gaalen, Susan Hahné, Hester de Melker, Susan van den Hof

**Affiliations:** 1 Centre for Infectious Disease Control, National Institute for Public Health and the Environment, Bilthoven, the Netherlands; 2 CBS, Statistics Netherlands, the Hague, the Netherlands; 3 University of Amsterdam, Amsterdam, the Netherlands; 4 Department of Clinical Epidemiology, Leiden University Medical Center, Leiden, the Netherlands

**Keywords:** Childhood vaccination, Pandemic impact, Coverage, COVID-19, DTaP-IPV, vaccination uptake

## Abstract

**BACKGROUND:**

The Netherlands has experienced a decline in routine childhood vaccination uptake during and after the COVID-19 pandemic, with emerging evidence of reduced parental vaccine confidence.

**AIM:**

To evaluate the immediate and long-term impact of the COVID-19 pandemic on first-dose diphtheria-tetanus-pertussis-poliomyelitis (DTaP-IPV) vaccination uptake.

**METHODS:**

We conducted a retrospective nationwide cohort study including all children born in the Netherlands in 2016–2024. First-dose DTaP-IPV vaccination status by age 6 months was obtained from the national immunisation register. National trends in vaccination uptake across pre-pandemic, pandemic and post-pandemic periods were assessed using interrupted time series analyses (ITS). To further assess the independent effects of the pandemic, matched-sibling analysis compared vaccination uptake within families before, during and after the pandemic.

**RESULTS:**

ITS showed significant immediate decreases in vaccination uptake both at the start and end of the pandemic, accompanied by a continuing downward trend during the pandemic (OR: 0.984; 95% CI: 0.981–0.986) that further declined after its end (OR: 0.995; 95% CI: 0.993–0.998). In the matched-sibling analysis, children eligible during and after the pandemic had lower odds of being vaccinated (pandemic: OR: 0.66; 95% CI: 0.55–0.80; post-pandemic: OR: 0.20; 95% CI: 0.17–0.25) compared with their pre-pandemic siblings. Also, later birth order was associated with lower odds compared with first-born siblings (second-born: OR: 0.42; 95% CI: 0.37–0.48).

**CONCLUSIONS:**

Both analyses indicate a negative impact of the COVID-19 pandemic on childhood vaccination uptake, which may reflect lingering pandemic effects and/or new post-pandemic factors, highlighting the need for further research into the drivers of vaccination uptake changes in the post-pandemic era.

**Key public health message**

**What did you want to address in this study and why?**

The COVID-19 pandemic disrupted routine healthcare services worldwide, including childhood vaccinations. High vaccination coverage is essential to protect children and communities from serious infectious diseases. We investigated the effect of the COVID-19 pandemic on routine childhood vaccination uptake in the Netherlands, specifically looking at the diphtheria-tetanus-pertussis-poliomyelitis (DTaP-IPV) vaccine, first administered at 2–3 months.

**What have we learnt from this study?**

We found that DTaP-IPV vaccination uptake was increasing slightly before the pandemic, from 95% for children born in 2016 to 96% for those born in 2019. During the pandemic, uptake declined to 93% for children born in 2021, and decreased further after the pandemic, with uptake falling to 89% for children born in 2024. Within families, children born during or after the pandemic were less likely to be vaccinated compared with siblings born beforehand.

**What are the implications of your findings for public health?**

The ongoing decline in childhood vaccination uptake following the COVID-19 pandemic increases the risk of outbreaks of vaccine-preventable diseases. Monitoring vaccination trends and understanding both existing and new barriers or concerns that may have emerged since the pandemic started are important for designing effective public health interventions to improve coverage.

## Introduction

The COVID-19 pandemic has disrupted routine healthcare services globally, including the delivery of childhood vaccinations [1]. In the Netherlands, the emergence of COVID-19 in February 2020 led to the rapid implementation of widespread public health measures. The Dutch government introduced a lockdown mid-March 2020. Alternating periods of stricter and more relaxed restrictions followed, before most measures were lifted early 2022 [2].

Although infant vaccination schedules were largely maintained, early data from the Netherlands indicated that the vaccination coverage for infants scheduled to receive their first measles-mumps-rubella (MMR1) vaccination between March and September 2020, was estimated to be 1–2 percentage points lower compared with the coverage among children born 1 year earlier [3]. Importantly, a decreasing trend in first-dose MMR and ≥ 3-dose diphtheria-tetanus-pertussis-poliomyelitis vaccination (DTaP-IPV) coverage (by age 2 years) was already apparent before the pandemic, although this decline was not consistent over the entire pre-pandemic period. Coverage, which had been consistently above 95% for the 2005–2011 birth cohorts, dropped to 92.6% for DTaP-IPV and 92.9% for MMR in cohort 2015. After a slight recovery to 93.1% (DTaP-IPV) and 93.3% (MMR) for the 2018 birth cohort, coverage declined again, reaching 87.9% and 87.7% for MMR and DTaP-IPV, respectively, in the 2022 birth cohort [4] (vaccination coverage from Dutch birth cohorts 2005–2022 is shown Supplementary Figure S1). It is important to note that, from 2022 onwards, a new policy in the Netherlands requires explicit parental informed consent for vaccination registration [4]. Consequently, children vaccinated without parental consent for registration are recorded as unvaccinated, which may partly account for the observed decrease in coverage in recent years [4].

While declining coverage was already observed before the pandemic, the pandemic may have introduced additional factors affecting vaccination uptake. The pandemic coincided with an unprecedented spread of mis- and disinformation and scepticism surrounding the SARS-CoV-2 virus, the public health measures and vaccines, particularly during the COVID-19 vaccine rollout [5]. Emerging evidence suggests that this infodemic has undermined trust not only in COVID-19 vaccines, but also in established childhood immunisation programmes. Studies indicate a decline in confidence and more negative attitudes among parents towards vaccination [6-8]. These findings raise the question whether the pandemic’s negative impact on vaccine coverage extends after it ended.

Therefore, this study aimed to assess the impact of the COVID-19 pandemic on first-dose DTaP-IPV vaccination uptake in the Netherlands, using national data from 2016 to 2025, using two complementary approaches. Firstly, we performed an interrupted time series analysis (ITS) to assess changes in population level vaccination trends and to determine whether the rate of decline in vaccine uptake changed at the start and at the end of the pandemic. Secondly, we use a matched-sibling analysis to compare siblings within the same family, thereby assessing the effect of the pandemic while controlling for family-specific factors that may influence vaccination decisions. Together, these analyses provide a comprehensive assessment of the pandemic’s impact on routine childhood vaccination in the Netherlands, by capturing both population-level trends and accounting for family-level differences in vaccination behaviour.

## Methods

### Study setting and population

This retrospective registry study was conducted using linked national data within the secure remote access environment of Statistics Netherlands (, CBS). We included children born 2016–2024, residing in the Netherlands on 31 December 2024, as registered in the personal records database. We excluded children who were not living in the Netherlands at time of eligibility for first-dose DTaP-IPV vaccination. In the Netherlands, the DTaP-IPV vaccination schedule for infants includes a first dose at 2 months of age for children born before 2020. Following the introduction of maternal pertussis vaccination in 2020, infants whose mothers received this vaccination are scheduled for their first dose at 3 months of age, while others continue to receive the first dose at 2 months.

### Categorisation of pandemic period and birth cohorts

Children were assigned to one of three pandemic periods based on their date of birth and the corresponding age of eligibility for their first-dose DTaP-IPV vaccination (Table 1). Pandemic periods were defined based on COVID-19-related public health measures in the Netherlands. Different measures were in place between March 2020 and January 2022, including social distancing, evening curfews and the closing of schools and public places. The relaxation of pandemic measures coincided with the introduction of informed consent of registration of vaccination in the national vaccination register Praeventis [9] on 1 January 2022. National data indicate that on average 1.7% of parents did not provide consent for primary series DTaP-IPV vaccination registration.

**Table 1:** Definitions of pandemic periods and corresponding birth cohorts for childhood vaccination uptake analysis, the Netherlands, 2016–2024

Period	Definition	Birth cohort
Pre-pandemic	Eligible for first-dose DTaP-IPV before implementation of pandemic response measures in Mar 2020	1 Jan 2016–31 Dec 2019
Pandemic	Eligible for vaccination during the period of COVID-19 measures and before the introduction of informed consent for national vaccination registration (Mar 2020–Jan 2022)	1 Jan 2020–30 Sep 2021
Post-pandemic	Eligible for vaccination after the relaxation of pandemic measures and following the introduction of the informed consent policy in Jan 2022	1 Oct 2021–31 Dec 2024

### Outcome measure

The primary outcome was having received the first-dose DTaP-IPV vaccination at the age of 6 months, to allow sufficient time for delayed vaccination beyond the scheduled age. A (meaningful) delay in vaccination was defined as not having received the first-dose DTaP-IPV vaccination by the age of 6 months. We examined the timing of vaccination in the vaccinated children in our study population, by birth year, and found that the majority of children were vaccinated in a timely manner, according to the schedule (median age range 2.5–3.5 months for birth cohorts 2016–2024) (see Supplementary Figure S2 for median age in months at time of first-dose DTaP-IPV vaccination by year of birth). Individual level vaccination status was retrieved from Praeventis on 28 August 2025.

### Interrupted time series analysis

We used an interrupted time series (ITS) design to assess changes in first-dose DTaP-IPV vaccination uptake. Interrupted time series is a quasi-experimental method for evaluating changes in outcomes following distinct intervention points or events [10]. Two interruptions were defined, corresponding to the transitions between the previously defined pandemic periods. We used individual-level data and segmented regression models to estimate changes in the probability of receiving the first scheduled DTaP-IPV vaccination associated with each interruption, capturing both immediate (level) and gradual (slope) changes over time. A binomial logistic regression model was used. Results are presented as odds ratios with 95% confidence intervals (CI), where each period is compared with the adjacent preceding period (i.e. the pandemic period is compared with the pre-pandemic period, and the post-pandemic period is compared with the pandemic period). To facilitate the interpretation of the resulting ORs, average monthly model predictions were used to calculate the absolute level changes. For the slopes, this is not possible; however, the direction of the slope, the change after the interruptions, and the statistical significance are more of interest than the exact value.

Firstly, a simple (unadjusted) model was created, including birth year and month, interruption 1 and 2 (level changes) and time since interruption 1 and 2 (slope changes):

Y_t_ = *β*_0_ + *β*_1_ (Time in months) + *β*_2_ (Interruption 1) + *β*_3_ (Time in months since interruption 1) + *β*_4_ (Interruption 2) + *β*_5_ (Time in months since interruption 2)

with Y_t_ being the chance of vaccination (on an individual level), *β*_0_ the intercept of the model, *β*_1_ the slope pre-pandemic, *β*_2_ the level change at the start of the pandemic, *β*_3_ the slope change since the start of the pandemic, *β*_4_ the level change at the end of the pandemic/start of informed consent, and *β*_5_ the slope change at the end of the pandemic/start of informed consent.

We extended the model by adjusting for individual-level sociodemographic variables available from CBS, including birth order, migration status (Dutch origin, migrant, child of first-generation migrant, child of second-generation migrant), maternal education level (‘high’: completed higher professional education (HBO) or university education (WO)) or ‘not high’ (primary education, pre-vocational education (VMBO), entry-level secondary vocational education (MBO1), senior general secondary education (HAVO), or pre-university education (VWO)), household income (quartiles based on total Dutch population), and urbanisation level based on the residential neighbourhood. Urbanisation level was defined according to the average address density per square kilometre as extremely urbanised (≥ 2,500 addresses/km^2^), strongly urbanised (1,500–2,499 addresses/km^2^), moderately urbanised (1,000–1,499 addresses/km^2^), hardly urbanised (500–999 addresses/km^2^), and not urbanised (≤500 addresses/km^2^). For descriptive statistics, we also included the variable country of origin. Because some categories of ‘country of origin’ and ‘migration status’ (e.g. ‘Dutch origin’ and ‘country of origin: the Netherlands’) overlapped completely, including both variables in the model would result in multicollinearity. Therefore, only migration status was included in the model, and country of origin was excluded from statistical analysis. The reference year for all sociodemographic variables was 2024. A more detailed description of the sociodemographic variables can be found in Supplementary Table S3.

Possible autocorrelation in the time series was assessed using the Durbin-Watson test and by visually inspecting the partial autocorrelation function (PACF) of the residuals.

### Matched-sibling analysis

We performed a matched-sibling analysis to improve the possibilities for causal inference by accounting for unmeasured family-level confounders, such as parental attitudes towards vaccination, socioeconomic status and other shared environmental or cultural factors. We included families with at least two children, with all children born in 2016–2024. Families in which any child was living outside the Netherlands at the time of first-dose DTaP-IPV vaccination eligibility were excluded. In the case of multiples, we included only the first child in the register, as vaccination decisions for multiples are typically made collectively and reflect a single family-level decision.

We employed conditional logistic regression to compare vaccination uptake among siblings within the same family, effectively matching on shared family-level factors. Birth order was included as an independent variable, as previous studies have shown that later-born children are at higher risk of incomplete or delayed vaccination [11]. We aimed to investigate whether this association persisted or changed during and after the pandemic. Therefore, we first constructed a model that included birth order and pandemic period as independent variables. In a second model, we added an interaction term between pandemic period and birth order to assess whether the effect of birth order on vaccination uptake differed before, during, and after the pandemic. In accordance with the principles of conditional logistic regression, only discordant families — those in which siblings differed in outcome — contributed to the estimation of model parameters. The same holds for the estimation of the effect of pandemic period; only discordant families with siblings eligible in different pandemic periods contributed information to these estimates [12]. Descriptive family-level characteristics were reported to provide context for generalisability of the findings.

### Sensitivity analyses

As a sensitivity analysis, we repeated the conditional logistic regression analysis restricted to families with two children to assess consistency and generalisability across different family sizes. This approach allows for a straightforward comparison between first- and second-born siblings and reduces potential heterogeneity associated with larger family sizes, which may differ in parental characteristics or vaccination behaviours [13]. Only families with siblings eligible in different pandemic periods contributed to estimating the effect of pandemic period. Because birth order and pandemic period overlapped in these families, interaction effects could not be estimated, so only the main effects model was used. To assess the potential impact of misclassification caused by incomplete parental consent for national vaccination registration after January 2022, we conducted a sensitivity analysis in which we reclassified a proportion of unvaccinated children eligible in the post-pandemic/informed consent period (2021–2024) as vaccinated. Specifically, since the 1.7% non-consent rate refers to the vaccinated population, we estimated the number of vaccinated children whose parents did not provide consent by proportionally adjusting the observed count: for every 98.3 children recorded as vaccinated, 1.7 vaccinated children are missing from the registry because of non-consent. This led to the formula: (number of vaccinated children in the subset/98.3) x 1.7, which estimates the number of unvaccinated children to be reclassified. For the ITS analysis, we did not apply this correction, as adjusting for non-consent would only shift the absolute coverage level in the post-pandemic period upward, without affecting the underlying slope over time. Therefore, the potential impact of incomplete consent on the ITS results is addressed in the interpretation of our findings rather than through direct adjustment of the estimates.

All analyses were performed in R version 4.4.3.

## Results

The total study population included 1,375,514 children of whom 1,284,440 (93%) had received a first-dose DTaP-IPV vaccination at 6 months of age (Table 2). Vaccination coverage was highest in the pre-pandemic period and declined slightly during and after the pandemic. Uptake was generally higher among first- and second-born children compared with later-born children. Children of Dutch origin had higher coverage than those of non-Dutch origin. Similarly, coverage was higher among children whose mother had a higher level of education and among those in households with higher income, compared with children whose mother had a lower level of education and those in households with lower income. Coverage also varied by country of origin and level of urbanisation.

**Table 2:** First-dose DTaP-IPV vaccination uptake in children at age 6 months by descriptive sociodemographic characteristics, the Netherlands, birth cohorts 2016–2024 (n = 1,375,514)

Characteristics	Total population		Vaccinated population	
	n	Column %	n	Row %
Total number of children	1,375,514	100	1,284,440	93
**Pandemic period **				
Pre-pandemic	589,003	43	562,205	95
Pandemic	277,223	20	260,742	94
Post-pandemic	509,288	37	461,493	91
**Birth order **				
First born	660,026	48	624,849	95
Second born	523,562	38	491,982	94
Third born	156,408	11	140,233	90
Fourth or later born	35,518	3	27,376	77
**Year of birth **				
2016	144,177	10	137,232	95
2017	145,791	11	139,035	95
2018	147,766	11	141,065	95
2019	151,269	11	144,873	96
2020	153,090	11	144,413	94
2021	165,515	12	154,694	93
2022	155,927	11	142,905	92
2023	154,422	11	139,444	90
2024	157,557	11	140,779	89
**Family size **				
One child	324,162	24	303,861	94
Two children	700,061	51	666,334	95
Three children	275,127	20	252,911	92
Four or more children	76,164	6	61,334	81
**Migration status **				
Dutch origin^a^	830,040	60	786,450	95
Migrant	3,370	< 1	2,797	83
Child of first-generation migrant(s)	332,893	24	309,386	93
Child of second-generation migrant(s)	209,211	15	185,807	89
**Country of origin^b^**				
Netherlands	830,040	60	786,450	95
Europe (excl. Netherlands)	152,629	11	143,539	94
Morocco	59,793	4	44,198	74
Turkey	51,613	4	44,796	87
Suriname	41,732	3	38,607	93
Dutch Caribbean	26,725	2	24,514	92
Indonesia	35,855	3	34,120	95
Other, America/Oceania	39,738	3	37,548	94
Other, Africa	47,652	3	44,793	94
Other, Asia	89,737	7	85,875	96
**Maternal education level^c^**				
High	658,273	48	624,970	95
Not high	717,241	52	659,470	92
**Household income^d^**				
First quartile	148,969	11	131,572	88
Second quartile	343,537	25	310,967	91
Third quartile	502,183	37	476,197	95
Fourth quartile	375,579	27	361,292	96
Unknown	5,246	< 1	4,412	84
**Level of urbanisation^e^**				
Not urbanised	216,838	16	200,647	93
Hardly urbanised	242,206	18	228,561	94
Moderately urbanised	264,172	19	249,176	94
Strongly urbanised	351,509	26	329,207	94
Extremely urbanised	300,313	22	276,409	92
Unknown	476	< 1	440	92

DTaP-IPV: diphtheria-tetanus-pertussis-poliomyelitis vaccine.

^a^ Dutch origin is defined as cases where the child, both parents, and all grandparents were born in the Netherlands.

^b^ Country of origin was determined hierarchically: first by the child’s country of birth; if the child was born in the Netherlands, then by the parents’ country of birth (prioritising the mother over the father); and if both parents were born in the Netherlands, then by the grandparents’ country of birth (prioritising the grandmother over the grandfather).

^c^ Maternal education level was categorised as ‘high’ (completed higher professional education (HBO) or university education (WO)) or ‘not high’ (primary education, pre-vocational education (VMBO), entry-level secondary vocational education (MBO1), senior general secondary education (HAVO), or pre-university education (VWO)).

^d^ Standardised disposable income (SDI) of the household. If parents of children were living in separate households, the average SDI of the two households was taken. Quartiles (percentiles) were calculated by Statistics Netherlands (CBS) based on the complete Dutch population. The first quartile represents the lowest income and the fourth quartile the highest.

^e^ Urbanisation level was defined according to the average address density per square kilometre as extremely urbanised (≥ 2,500 addresses/km^2^), strongly urbanised (1,500–2,499 addresses/km^2^), moderately urbanised (1,000–1,499 addresses/km^2^), hardly urbanised (500–999 addresses/km^2^), and not urbanised (≤500 addresses/km^2^).

### Interrupted time series analysis

The unadjusted ITS model, taking into account only year and month of birth, showed a slightly increasing trend in vaccination uptake pre-pandemic (OR: 1.004, 95% CI: 1.003–1.004) (Table 3). After the start of the pandemic, an initial significant drop in vaccination uptake was found with an OR of 0.777 (95% CI: 0.746–0.809). This corresponds to an absolute level change of −1.12 percent points. Simultaneously, the increasing trend in vaccination uptake that existed pre-pandemic significantly decreased after the start of the pandemic (OR: 0.985, 95% CI: 0.983–0.988). At the end of the pandemic, a significant level change was observed (OR: 0.868, 95% CI: 0.836–0.900), corresponding to an absolute level change of −1.00 percent points. This should, however, be interpreted with caution, as the timepoint used for the end of the pandemic coincided with the start of informed consent for vaccination registration. No significant post-pandemic slope change was found compared with the pandemic period (OR: 0.999, 95% CI: 0.996–1.002). Regarding potential residual autocorrelation, the Durbin-Watson statistic was 1.43 (p < 0.001), however, the PACF did not show a clear or systematic autocorrelation structure.

**Table 3:** Interrupted time series model results for first-dose DTaP-IPV vaccination uptake at age 6 months, unadjusted (n = 1,375,514) and adjusted for sociodemographic factors (n = 1,369,801), the Netherlands, birth cohort 2016–2024

Characteristics	Unadjusted model			Adjusted model		
	OR	95% CI	p value	OR	95% CI	p value
Slope (birth month)	1.004	1.003–1.004	< 0.001	1.006	1.005–1.007	< 0.001
Level change: start pandemic^a^	0.777	0.746–0.809	< 0.001	0.765	0.735–0.798	< 0.001
Slope change: start pandemic^a^	0.985	0.983–0.988	< 0.001	0.984	0.981–0.986	< 0.001
Level change: end pandemic/start informed consent^b^	0.868	0.836–0.900	< 0.001	0.896	0.863–0.930	< 0.001
Slope change: end pandemic/start informed consent^b^	0.999	0.996–1.002	0.558	0.995	0.993–0.998	0.001
Birth order						
First born	NA			Ref.		
Second born				0.907	0.892–0.921	< 0.001
Third born				0.552	0.541–0.563	< 0.001
Fourth born or later				0.256	0.249–0.264	< 0.001
Migration status						
Dutch origin^c^	NA			Ref.		
Migrant				0.306	0.278–0.336	< 0.001
Child of first-generation migrant(s)				0.926	0.909–0.943	< 0.001
Child of second-generation migrant(s)				0.467	0.459–0.475	< 0.001
Maternal education level^d^						
High	NA			Ref.		
Not high				0.864	0.850–0.877	< 0.001
Household income^e^						
Fourth quartile	NA			Ref.		
Third quartile				0.700	0.685–0.715	< 0.001
Second quartile				0.395	0.387–0.404	< 0.001
First quartile				0.347	0.339–0.356	< 0.001
Level of urbanisation^f^						
Not urbanised	NA			Ref.		
Hardly urbanised				1.352	1.320–1.385	< 0.001
Moderately urbanised				1.418	1.385–1.452	< 0.001
Strongly urbanised				1.357	1.327–1.388	< 0.001
Extremely urbanised				1.151	1.125–1.178	< 0.001

CI: confidence interval; DTaP-IPV: diphtheria-tetanus-pertussis-poliomyelitis vaccine; NA: not applicable; OR: odds ratio; Ref.: reference.

^a^ Reference period: pre-pandemic (birth cohort 1 Jan 2016–31 Dec 2019).

^b^ Reference period: pandemic (birth cohort 1 Jan 2020–30 Sep 2021).

^c^ Dutch origin is defined as cases where the child, both parents, and all grandparents were born in the Netherlands.

^d^ Maternal education level was categorised as ‘high’ (completed higher professional education (HBO) or university education (WO)) or ‘not high’ (primary education, pre-vocational education (VMBO), entry-level secondary vocational education (MBO1), senior general secondary education (HAVO), or pre-university education (VWO)).

^e^ Standardised disposable income (SDI) of the household. If parents of children were living in separate households, the average SDI of the two households was taken. Quartiles (percentiles) were calculated by Statistics Netherlands (CBS) based on the complete Dutch population. The first quartile represents the lowest income and the fourth quartile the highest.

^f^ Urbanisation level was defined according to the average address density per square kilometre as extremely urbanised (≥ 2,500 addresses/km^2^), strongly urbanised (1,500–2,499 addresses/km^2^), moderately urbanised (1,000–1,499 addresses/km^2^), hardly urbanised (500–999 addresses/km^2^), and not urbanised (≤500 addresses/km^2^).

The p values indicate the statistical significance of the association between each characteristic and first-dose DTaP-IPV vaccination uptake and, for level and slope changes, whether these changes differ significantly from the underlying trend; p < 0.05 was considered statistically significant.

After adjusting for sociodemographic factors, similar statistically significant decreases in vaccination uptake were seen directly after both interruptions (OR: 0.765; 95% CI: 0.735–0.798) and 0.896 (95% CI: 0.863–0.930) at the start and end of the pandemic, as can be seen in Table 3 and the Figure. These correspond to level changes of −1.15 and −0.66 percent points at the start and end of the pandemic, respectively. Again, the level change should be interpreted in the light of the implementation of informed consent. After the start of the pandemic, a significant slope change was found (OR: 0.984; 95% CI: 0.981–0.986) compared with pre-pandemic, indicating a decreasing trend in vaccination uptake. Opposed to the unadjusted model, in the adjusted model we did find a statistically significant slope change after the end of the pandemic compared with during the pandemic, with an OR of 0.995 (95% CI: 0.993–0.998). This indicates that the already decreasing vaccination uptake observed during the pandemic dropped at an even faster rate after the end of the pandemic. Furthermore, when comparing the vaccination uptake (Table 2) to the adjusted estimates of the covariates in the adjusted model, similar trends in vaccination uptake are seen across specific subgroups. 

**Figure:**
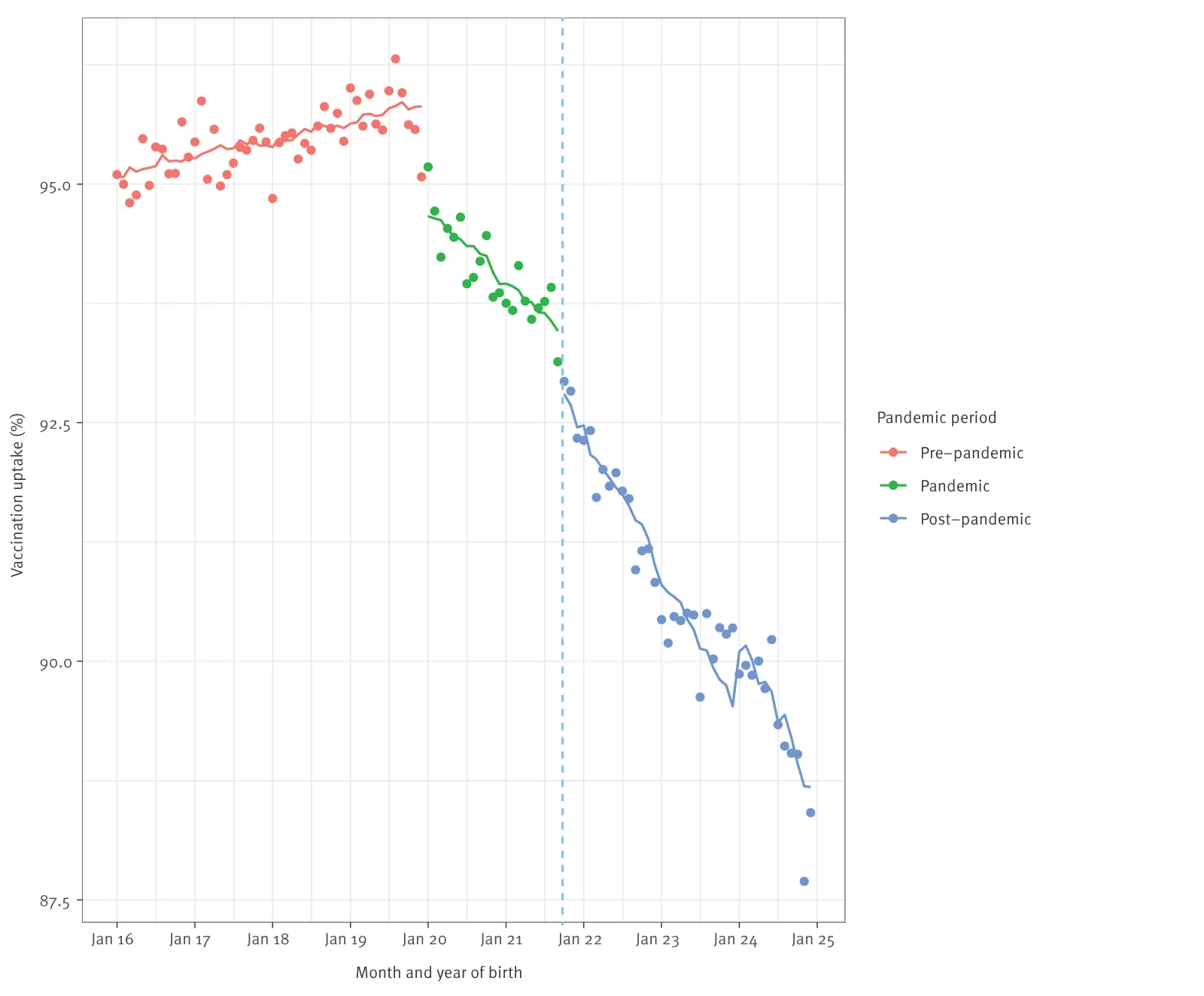
Vaccination coverage for DTaP-IPV in children at age 6 months, by pandemic period, the Netherlands, birth cohorts 2016–2024 (n = 1,369,801)

DTaP-IPV: diphtheria-tetanus-pertussis-poliomyelitis vaccine.

Children born in January 2020 were eligible for the first dose of DTaP-IPV vaccine in March 2020 (start of the pandemic), and children born in October 2021 were eligible in January 2022 (end of the pandemic/start of informed consent). The points represent observed vaccine uptake by months and year of birth, and lines indicate the adjusted model prediction. The model predictions are not linear, as they are based on the average predicted vaccination uptake per month, e.g. predictions are made on an individual level, and then an average predicted vaccination uptake per month is calculated.

### Matched-sibling analysis

Table 4 presents the descriptive characteristics of concordant and discordant families included in the matched-sibling analysis. The study population included 708,059 children in 326,981 families. The majority of families were concordant for vaccination status, with 301,465 families (92%) having all eligible children vaccinated and 13,164 families (4%) having all children unvaccinated. The remaining 12,352 families (4%) were discordant, meaning siblings differed in vaccination status and thus contributed information to the conditional logistic regression. Most families had two children (86% of concordant-all-vaccinated, 74% of concordant-all-unvaccinated, and 68% of discordant families). Discordant families were more often of non-Dutch origin, had a lower maternal education level, and lived in more urbanised areas compared with concordant families, particularly compared with concordant families in which all children were vaccinated.

**Table 4:** Family-level characteristics by sibling vaccination concordance for first-dose DTaP-IPV vaccination uptake at age 6 months including families with at least two children born 2016–2024, the Netherlands (n = 326,981)

Characteristics	Concordant families				Discordant families	
	All vaccinated		All unvaccinated			
	n	Column %	n	Column %	n	Column %
Total	301,465	100	13,164	100	12,352	100
Family size						
Two children	259,145	86	9,695	74	8,408	68
Three children	39,573	13	2,780	21	3,341	27
Four or more children	2,747	1	689	5	603	5
Migration status						
Dutch origin^a^	199,511	66	6,649	51	4,755	38
Migrant	585	< 1	63	< 1	61	< 1
Child of first-generation migrant(s)	58,171	19	2,876	22	3,721	30
Child of second-generation migrant(s)	43,198	14	3,576	27	3,815	31
Country of origin^b^						
Netherlands	199,511	66	6,649	51	4,755	38
Europe (excl. the Netherlands)	30,346	10	1,126	9	1,184	10
Morocco	8,142	3	2,454	19	2,419	20
Turkey	9,028	3	972	7	1,437	12
Suriname	7,752	3	343	3	516	4
Dutch Caribbean	4,837	2	263	2	344	3
Indonesia	7,869	3	223	2	240	2
Other, America/Oceania	8,179	3	287	2	273	2
Other, Africa	8,825	3	347	3	527	4
Other, Asia	16,687	6	500	4	657	5
Maternal education level^c^						
High	159,949	53	4,820	37	4,487	36
Not high	141,516	47	8,344	63	7,865	64
Household income^d^						
First quartile	24,580	8	2,441	19	2,260	18
Second quartile	70,417	23	4,930	37	4,269	35
Third quartile	121,283	40	3,877	29	3,836	31
Fourth quartile	84,624	28	1,831	14	1,913	15
Unknown	561	< 1	85	< 1	74	< 1
Level of urbanisation^e^						
Not urbanised	50,879	17	2,599	20	1,564	13
Hardly urbanised	57,642	19	2,080	16	1,685	14
Moderately urbanised	60,669	20	2,239	17	2,010	16
Strongly urbanised	75,398	25	3,121	24	3,340	27
Extremely urbanised	56,776	19	3,121	24	3,747	30
Unknown	101	< 1	< 10	< 1	< 10	< 1

DTaP-IPV: diphtheria-tetanus-pertussis-poliomyelitis vaccine.

^a^ Dutch origin is defined as cases where the child, both parents, and all grandparents were born in the Netherlands.

^b^ Country of origin was determined hierarchically: first by the child’s country of birth; if the child was born in the Netherlands, then by the parents’ country of birth (prioritising the mother over the father); and if both parents were born in the Netherlands, then by the grandparents’ country of birth (prioritising the grandmother over the grandfather).

^c^ Maternal education level was categorised as ‘high’ (completed higher professional education (HBO) or university education (WO)) or ‘not high’ (primary education, pre-vocational education (VMBO), entry-level secondary vocational education (MBO1), senior general secondary education (HAVO), or pre-university education (VWO)).

^d^ Standardised disposable income (SDI) of the household. If parents of children were living in separate households, the average SDI of the two households was taken. Quartiles (percentiles) were calculated by Statistics Netherlands (CBS) based on the complete Dutch population. The first quartile represents the lowest income and the fourth quartile the highest. This characteristics table provides context for understanding the analytic sample; however, by design, the within-family analysis controls for stable family-level factors, and thus these differences do not confound the estimated effects within discordant families. All reported tables exclude frequencies below ten to avoid personally identifiable information.

^e^ Urbanisation level was defined according to the average address density per square kilometre as extremely urbanised (≥2,500 addresses/km^2^), strongly urbanised (1,500–2,499 addresses/km^2^), moderately urbanised (1,000–1,499 addresses/km^2^), hardly urbanised (500–999 addresses/km^2^), and not urbanised (≤500 addresses/km^2^).

Table 5 presents the results of the matched-sibling conditional logistic regression models. In Model 1, both pandemic period and birth order were independently associated with vaccination uptake within discordant families. Children who became eligible for vaccination during the pandemic had lower odds of receiving the first-dose DTaP-IPV vaccination compared with their siblings eligible before the pandemic (OR: 0.50; 95% CI: 0.44–0.55), and this effect was even more pronounced for those eligible after the pandemic (OR: 0.15; 95% CI: 0.13–0.18). Birth order also showed a strong association with vaccination uptake: compared with first-born siblings, the odds of vaccination were lower for second-born (OR: 0.32, 95% CI: 0.29–0.35), third-born (OR: 0.14, 95% CI: 0.12–0.16), and fourth or later-born children (OR: 0.08, 95% CI: 0.06–0.10).

**Table 5:** Matched-sibling conditional logistic regression model for first-dose DTaP-IPV vaccination uptake at age 6 months including children born 2016–2024 within families with at least two children, the Netherlands (n = 326,981; n discordant = 12,352)

**Characteristics**	**Model 1**			**Model 2**		
	**OR**	**95% CI**	**p value**	**OR**	**95% CI**	**p value**
Pre-pandemic	Ref.			Ref.		
Pandemic	0.50	0.44–0.55	< 0.001	0.66	0.55–0.80	< 0.001
Post-pandemic	0.15	0.13–0.18	< 0.001	0.20	0.17–0.25	< 0.001
First born	Ref.			Ref.		
Second born	0.32	0.29–0.35	< 0.001	0.42	0.37–0.48	< 0.001
Third born	0.14	0.12–0.16	< 0.001	0.19	0.10–0.35	< 0.001
Fourth or later born	0.08	0.06–0.10	< 0.001	0.07	0.00–3.75	0.193
Pandemic * second born	NA			0.53	0.42–0.68	< 0.001
Post-pandemic * second born				0.66	0.55–0.79	< 0.001
Pandemic * third born				0.64	0.33–1.24	0.182
Post-pandemic * third born				0.60	0.32–1.13	0.117
Pandemic * fourth or later born				0.43	0.01–24.65	0.682
Post-pandemic * fourth or later born				0.94	0.02–47.86	0.973

CI: confidence interval; DTaP-IPV: diphtheria-tetanus-pertussis-poliomyelitis vaccine; NA: not applicable; OR: odds ratio; Ref.: reference.

The p values indicate the statistical significance of the association between each characteristic and first-dose DTaP-IPV vaccination uptake; for interaction terms, p values indicate whether the association between birth order and vaccination uptake differed by pandemic period, with p < 0.05 considered statistically significant.

In Model 2, the effect of birth order on vaccination uptake was modified by the pandemic period. For second-born children, the interaction terms were statistically significant, indicating lower odds of vaccination during and after the pandemic compared with first-born children (OR: 0.53; 95% CI: 0.42–0.68 and OR: 0.66; 95% CI: 0.55–0.79 for pandemic and post-pandemic, respectively). For third-born and fourth- or later-born children, the interaction terms were not statistically significant. The total effects of birth order and pandemic period on vaccination uptake, calculated relative to first-born children eligible for vaccination pre-pandemic, are presented in Supplementary Table S4.

### Sensitivity analysis

We first repeated the matched-sibling conditional logistic regression analysis (model 1), restricting the sample to families with exactly two children (n = 277,248, discordant n = 8,408). Results were nearly identical to those of the main analysis (see Supplementary Table S5 for the model outcomes).

Additionally, we conducted a sensitivity analysis to address incomplete parental consent in the post-pandemic/informed consent period by reclassifying 16% of unvaccinated children as vaccinated. The results remained broadly consistent with the primary analysis, although the effect sizes for birth order were smaller and the interaction between being second-born and the post-pandemic period was not statistically significant (see Supplementary Tables S6 and S7 for the model outcomes of the sensitivity analysis).

## Discussion

This study assessed the impact of the COVID-19 pandemic on routine childhood vaccination uptake using two complementary approaches. Both analyses consistently showed a negative effect of the pandemic on first-dose DTaP-IPV uptake, with a sustained decline continuing post-pandemic (89% in birth cohort 2024). This sustained decrease may reflect lingering pandemic effects or new post-pandemic factors, and is concerning for increased risk of vaccine-preventable disease outbreaks and weakened herd immunity.

The ITS analysis revealed both a downward trend and abrupt level changes in vaccine uptake, coinciding with the start of the pandemic and the end of the pandemic and start of informed consent policy. The initial drop is likely due to pandemic-related disruptions and public health measures, as previously observed for MMR1 vaccination in the Netherlands [3]. The second interruption at the end of the pandemic coincided with the introduction of informed consent for vaccination registration. Although the estimated level change from our model (−1.00 percentage points in the unadjusted model, −0.66 percentage points in the adjusted model) is a bit smaller than the estimated proportion of vaccinated individuals for whom informed consent is lacking (1.7%), the abrupt decline at this time point is most likely explained by this policy change. However, we expect no impact of the implementation of informed consent on the slope parameter. The matched-sibling approach adds value by controlling for family-level factors (such as parental attitudes towards vaccination and cultural factors), better isolating the independent effect of the pandemic from other potential confounding factors, including trends in the composition of the population.

Our findings are consistent with evidence from other European countries, such as Italy and the United Kingdom (UK), reporting reduced childhood vaccination uptake during the pandemic, often falling below the World Health Organization (WHO) target of 90% [14-17]. While some recovery has been observed, declines persistent and socioeconomic disparities have widened [16,17]. Most studies, however, focus on the short-term impact; longer-term effects remain less clear. Outside of Europe, similar effects have been reported, although local factors such as healthcare system disruptions may influence the magnitude and duration of impact [18,19]. These patterns underline the importance of ongoing monitoring of vaccination uptake and further study to address lower vaccination uptake.

The sustained decrease in vaccination uptake post-pandemic suggests lingering pandemic effects or new post-pandemic contributing factors. A frequently mentioned outcome of the pandemic is a decrease in vaccine confidence [6]. Studies from Flanders (Belgium) and the UK indicate that, while most parents remain confident in childhood vaccines, overall confidence has decreased since the pandemic, especially among ethnic minority groups, which now express more concerns than before [20-23]. In addition, a systematic review has shown that the pandemic’s impact on vaccine confidence varies by context and may change over time [24]. In the Netherlands, while coverage remains relatively high compared with other European countries, parental vaccine confidence appears to have decreased after the pandemic [7]. Contributing factors likely include the widespread circulation of misinformation, especially via social media, which has shaped negative attitudes [5,25-27], reduced trust in governmental institutions caused by rapidly changing policies and restrictive measures, and concerns about the rapid development and rollout of COVID-19 vaccines [5]. Importantly, vaccine confidence has also been declining in some contexts independent of the pandemic, with increasing politicisation of vaccination debates and greater polarisation in public discourse potentially affecting broader perceptions of authority and public health initiatives [28]. To address these challenges, public health authorities should strengthen efforts to rebuild vaccine confidence, counter misinformation, and engage communities through targeted communication and outreach initiatives

Several studies report that later-born children are at higher risk of incomplete or delayed immunisation compared with first-born children, possibly on account of parental time constraints or changing perceptions [11,29]. This is in line with our findings and is concerning because, if this pattern of declining vaccine uptake over time persists, children born later into families currently represented by only one child may be less likely to receive a vaccination. However, the association was not statistically significant for fourth or later-born children, likely due to the small number of families with discordant vaccination status in this group, which limited statistical power. These findings underscore the importance of awareness among healthcare professionals that vaccination of an older sibling does not guarantee uptake for subsequent children, and highlight the continued need to provide vaccination information to all parents, regardless of previous vaccination history.

A key strength of this study is the use of the two complementary methodological approaches, enhancing validity and interpretability of our findings by providing both a broad epidemiological perspective and a more refined within-family comparison. Notably, both analyses consistently indicated a negative effect on DTaP-IPV vaccine uptake since the pandemic, lending greater confidence to the observed association. Furthermore, the use of nationwide, registry-based data enabled a comprehensive assessment of vaccination trends and adjustment for key sociodemographic factors. Lastly, by using recent data extending through 2025, we were able to evaluate both immediate and longer-term effects after the COVID-19 pandemic on childhood vaccination uptake, in contrast to earlier studies that have largely been limited to short-term outcomes.

Several limitations should be considered. Firstly, post-pandemic vaccination coverage is likely underestimated because of incomplete registration following the introduction of informed consent; for primary DTaP-IPV, the average percentage of parents not providing consent is relatively low (1.7% compared with compared with over 3–10% for DTP booster and school-age vaccinations) [4]. Although sensitivity analyses still showed a negative effect, variation in consent rates across subgroups may still have introduced bias, which we were unable to adjust for due to lack of data. Secondly, while the ITS controlled for pre-existing trends, unmeasured confounding remains possible given concurrent pandemic-related factors. In addition, sociodemographic variables were measured at a single time point (2024) and may not reflect changes over the study period, though major shifts are unlikely as migration variables and maternal education level remain stable after the birth of the first child and household income and level of urbanisation typically do not change dramatically for most families between births or subsequent children. Thirdly, children who died or moved abroad before 31 December 2024 were excluded, slightly underrepresenting the at-risk population; however, the impact is minimal given low childhood mortality and relatively low emigration rates among young children. Fourthly, some children may have received their vaccination later than usual because of pandemic delays; however, assessing vaccination status at 9 months of age showed only minor increase in coverage (0.2 percentage points) and no differences between pandemic periods (data not shown), indicating delays were not substantial [3]. Furthermore, the classification of children into pandemic periods by month of eligibility means some children born around period transitions might be vaccinated in a different pandemic period than the one they were assigned to. Since most children are vaccinated close to the time they are eligible (Supplementary Figure S2), this will only apply to a small number of children and we expect no impact on the results. Although the Durbin–Watson test suggests possible autocorrelation, the absence of an autocorrelation pattern in the residuals, combined with the cross-sectional nature of the individual-level data and adjustment for calendar time in the model, suggests that autocorrelation is very unlikely to materially affect the estimated standard errors or statistical inference of the ITS models. Finally, the matched-sibling analysis estimates effects only within families where siblings differ in both vaccination status and pandemic period, which may limit the generalisability [12]. Covariates associated with lower vaccination coverage may not hold true for one-child families, and the results could be influenced by unmeasured confounding, such as changes in family circumstances (e.g. divorce or household composition). However, only 5% of households in this analysis included siblings with different parents (0.4% different mother; 4.6% different father), and excluding these households yielded nearly identical results (data not shown). Analysis restricted to families with exactly two children also produced similar findings, supporting the robustness of our results.

## Conclusion

This nationwide study demonstrates a sustained decline in routine childhood vaccination uptake in the Netherlands during the COVID-19 pandemic that continued after the pandemic. Both population-level and within-family analyses indicate that the pandemic has had a negative impact on childhood vaccination uptake, which may reflect lingering pandemic effects or new post-pandemic factors. The observation that the downward trend in vaccination coverage continues rather than stabilises, is particularly concerning as it may increase the risk of outbreaks of vaccine-preventable diseases and undermine herd immunity. Therefore, continuous monitoring of vaccination uptake and further research is essential to understand changes in practical barriers to and attitudes towards vaccination, alongside continued efforts to manage mis- and disinformation and address vaccine hesitancy.

## Data Availability

All data are available within CBS Microdata and can be made available under strict conditions.

## Supplementary Data

396000 Supplement
