# Supplementary material for: Assessing the impact of the COVID-19 pandemic on routine childhood vaccination uptake, the Netherlands, 2016 to 2025
Source: Euro Surveill. 2026 Oct 8;31(40):2600159. doi: 10.2807/1560-7917.ES.2026.31.40.2600159 (PMC13653212; doi:10.2807/1560-7917.ES.2026.31.40.2600159)
Supplement: Supplementary file 1 [file 26-00159_PIJPERS_Supplement.pdf]

Supplementary material

This supplementary material is hosted by Eurosurveillance as supporting information alongside the article 'Assessing the impact of the COVID-19 pandemic on routine childhood vaccination uptake, The Netherlands, 2016-2025', on behalf of the authors, who remain responsible for the accuracy and appropriateness of the content. The same standards for ethics, copyright, attributions and permissions as for the article apply. Supplements are not edited by Eurosurveillance and the journal is not responsible for the maintenance of any links or email addresses provided therein.

Figure S1. First-dose MMR and ≥ 3 dose DTaP-IPV vaccination coverage at age 2 years, birth cohort 2005-2022, The Netherlands. Data retrieved from the annual vaccination coverage report by the Dutch National Public Health Institute [4].

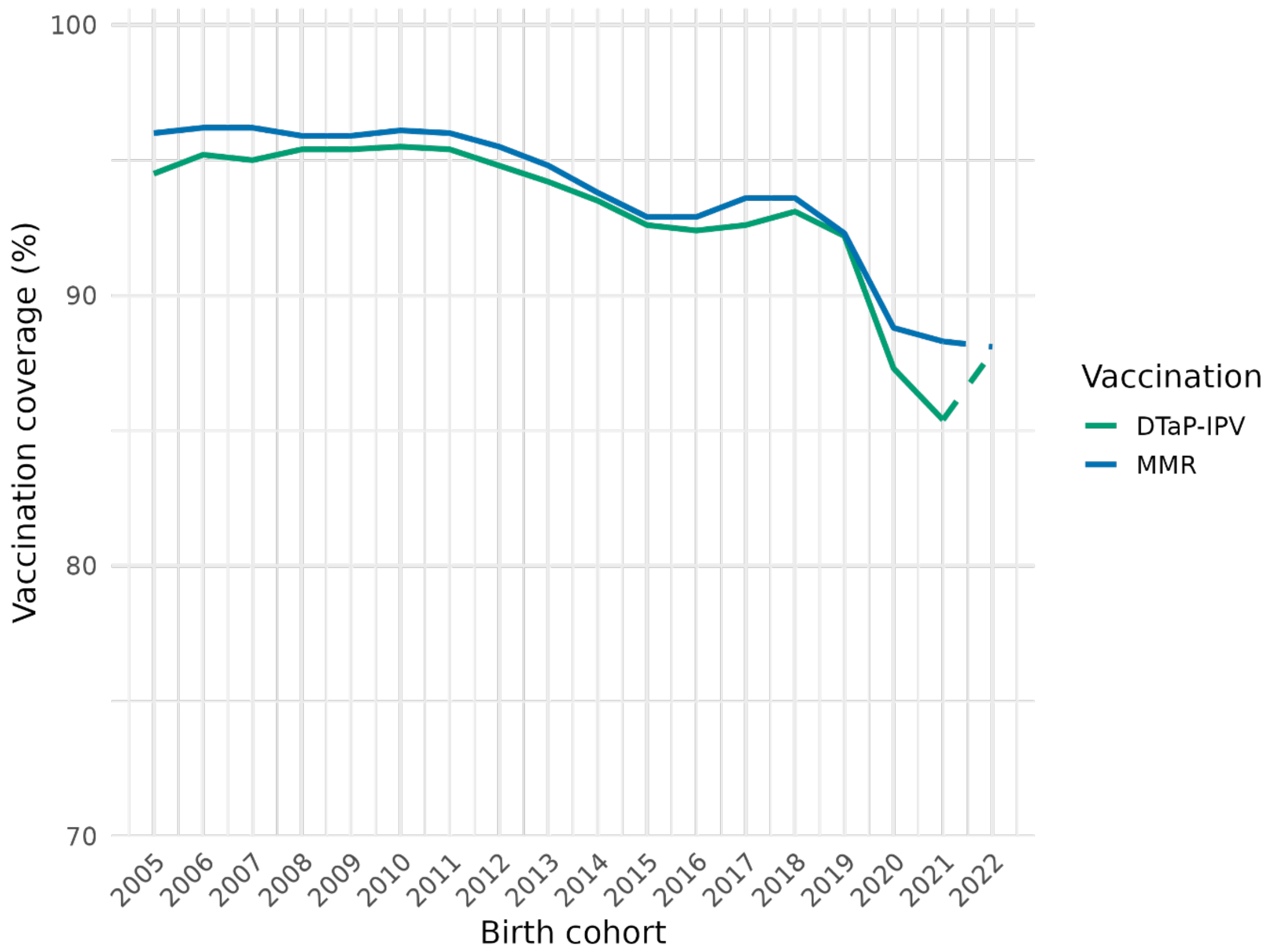

Abbreviations: *DTaP-IPV* diphtheria-tetanus-pertussis-polio, *MMR* measles-mumps-rubella
The dashed lines indicate the vaccination coverage is impacted by the informed consent: since January 2022 informed consent is required for registration of vaccination data in the national immunization register. Note: changes in registered vaccination coverage in the most recent cohort do not necessarily reflect changes in actual coverage, as recent figures do not yet account for anonymous vaccinations or administrative corrections for missing indications. For DTaP-IPV, the apparent increase in coverage in cohort 2025 is due to such an administrative correction and does not indicate a true rise in uptake; actual coverage is likely slightly lower than the previous year.

Figure S2. Median age in months at time of first-dose DTaP-IPV vaccination in children born 2016-2024 by year of birth (n = 1,375,514). Displayed are medians with interquartile range. Minimum and maximum values and outliers were removed from the graph to avoid identifiable information. Since birth year was unknown and therefore all children were coded as born on the 1st of the month, the true medians are slightly lower.

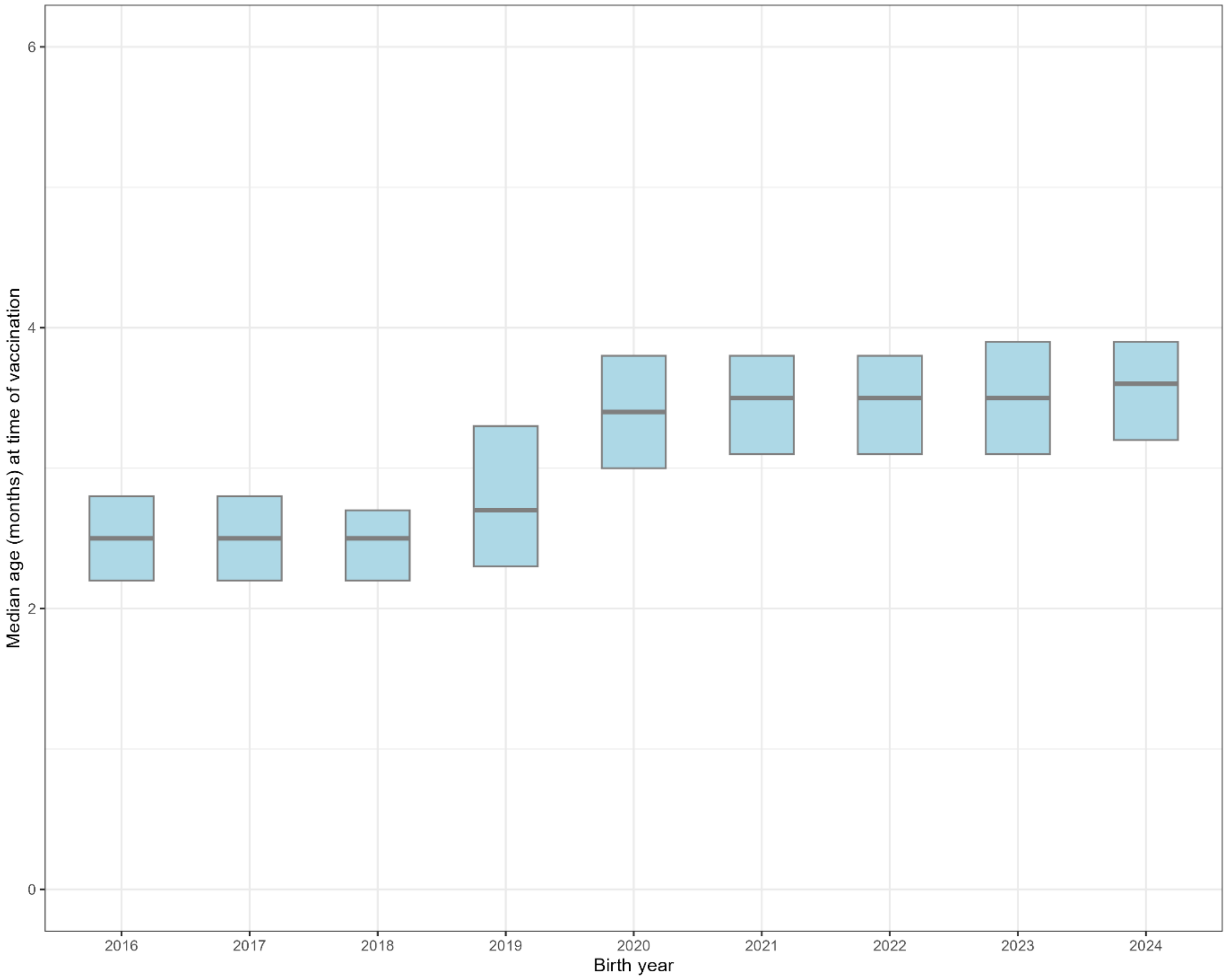

Table S3. Sociodemographic variables definitions

Variable	Definition	Levels	Reference year
Migration status	Migrations status based on whether: 1) children were born in the Netherlands, 2) parents were born in the Netherlands and 3) grandparents were born in the Netherlands	Dutch origin; first-generation migrant; child of first-generation migrant; child of second-generation migrants	2024
Country of origin	Country of origin was primarily defined by the country of birth (CoB) of the child itself. If the child was born in the Netherlands, country of origin was first determined by the parents' country of birth (CoB). If both parents were born in the Netherlands, the grandparents' CoB was then considered. Within this definition, the mother's CoB was prioritized over the father's, and the grandmother's CoB was prioritized over the grandfather's. Foreign-born origins were given precedence over Dutch origin if present in the lineage.	The Netherlands; Europe (excluding the Netherlands); Morocco; Turkey; Surinam; Dutch Caribbean; Indonesia; Other, America/Oceania; Other, Africa; Other, Asia). Indonesia; Other (Africa, Asia, America/Oceania)	2024
Maternal education level	Highest level of completed education. High: higher professional education (HBO); University education (WO) Not high: primary education; pre-vocational education	High; not high	2024

	(VMBO); entry-level secondary vocational education;<br>senior general secondary education<br>pre-university education		
Household standardised disposable income	Disposable income of the household taking into account differences in household size and household composition, divided into quartiles based on the entire Dutch population.	First quartile (lowest); second quartile; third quartile; fourth quartile (highest); unknown	2024
Level of Urbanisation	Urbanisation level was defined based on the average address density per square kilometre: extremely urbanised (≥2500 addresses/km2), strongly urbanised (1500-2499 addresses/km2), moderately urbanised (1000-1499 addresses/km2), hardly urbanised (500-999 addresses/km2) and not urbanised (≤500 addresses/km2).	Non-urbanised; hardly urbanised; moderately urbanised; strongly urbanised; extremely urbanised; unknown	2024

Table S4. Total effects of birth order and pandemic period on first-dose DTaP-IPV vaccination uptake among children in families with at least two children (n = 326,981; discordant n = 12,352). Reference group: first-born child, pre-pandemic period.

Group (ref: first child pre-pandemic)	OR	95%CI
Second child pandemic	0.15	0.11-0.21
Second child post pandemic	0.06	0.04-0.08
Third child pandemic	0.08	0.03-0.20
Third child post pandemic	0.02	0.01-0.06
Fourth or later child pandemic	0.02	0.00-5.94
Fourth or later child post pandemic	0.01	0.00-3.68

Abbreviations: *DTaP-IPV* diphtheria-tetanus-pertussis-polio

Table S5. Matched-sibling conditional logistic regression model for first-dose DTaP-IPV vaccination uptake at age 6 months, children born 2016-2024 within families with two children (n = 277,248; n discordant = 8,408)

	**OR**	**95% CI**	**p value**
Pre-pandemic (ref)	ref	ref	ref
Pandemic	0.50	0.43-0.58	<0.001
Post pandemic	0.14	0.12-0.18	<0.001
First born (ref)	ref	ref	ref
Second born	0.32	0.29-0.35	<0.001

Abbreviations: *DTaP-IPV* diphtheria-tetanus-pertussis-polio

Table S6. Sensitivity analysis with 16% of unvaccinated children in the post-pandemic/informed consent period reclassified as vaccinated. Matched-sibling conditional logistic regression model for first-dose DTaP-IPV vaccination uptake at age 6 months, children born 2016–2024 within families with at least two children (n = 326,981; n discordant = 12,352)

	Model 1			Model 2		
	OR	95% CI	p value	OR	95% CI	p value
Pre-pandemic (ref)	ref	ref	ref	ref	ref	ref
Pandemic	0.43	0.40-0.47	<0.001	0.48	0.43-0.54	<0.001
Post pandemic	0.35	0.32-0.40	<0.001	0.36	0.32-0.41	<0.001
First born (ref)	ref	ref	ref	ref	ref	ref
Second born	0.49	0.46-0.52	<0.001	0.53	0.47-0.58	<0.001
Third born	0.34	0.30-0.39	<0.001	0.31	0.19-0.51	<0.001
Fourth or later born	0.40	0.32-0.50	<0.001	0.21	0.01-4.62	0.320
pandemic* second born				0.82	0.69-0.98	0.026
post pandemic * second born				0.96	0.84-1.11	0.584
pandemic * third born				0.90	0.53-1.51	0.683
post pandemic * third born				1.14	0.69-1.87	0.616
pandemic * fourth or later born				0.51	0.02-13.03	0.687
post pandemic * fourth or later born				2.01	0.09-45.14	0.661

Abbreviations: *DTaP-IPV* diphtheria-tetanus-pertussis-polio

Table S7. Total effects of birth order and pandemic period on first-dose DTaP-IPV vaccination uptake among children in families with at least two children (sensitivity analysis reclassifying 16% of unvaccinated children in the post-pandemic/informed consent period as vaccinated (n = 326,981; n discordant = 12,352). Reference group: first-born child, pre-pandemic period.

Group (ref: first child pre-pandemic)	OR	95%CI
Second child pandemic	0.21	0.11-0.21
Second child post pandemic	0.18	0.04-0.08
Third child pandemic	0.13	0.03-0.20
Third child post pandemic	0.13	0.01-0.06
Fourth or later child pandemic	0.05	0.00-5.94
Fourth or later child post pandemic	0.15	0.00-3.68

Abbreviations: *DTaP-IPV* diphtheria-tetanus-pertussis-polio
